# Pioglitazone is as effective as dexamethasone in a cockroach allergen-induced murine model of asthma

**DOI:** 10.1186/1465-9921-8-90

**Published:** 2007-12-04

**Authors:** Venkata R Narala, Rajesh Ranga, Monica R Smith, Aaron A Berlin, Theodore J Standiford, Nicholas W Lukacs, Raju C Reddy

**Affiliations:** 1 Department of Internal Medicine, Division of Pulmonary and Critical Care Medicine, University of Michigan Medical Center, Ann Arbor, MI 48109-2200, USA; 2 Department of Pathology, University of Michigan, Ann Arbor, MI 48109, USA

## Abstract

**Background:**

While glucocorticoids are currently the most effective therapy for asthma, associated side effects limit enthusiasm for their use. Peroxisome proliferator-activated receptor-γ (PPAR-γ) activators include the synthetic thiazolidinediones (TZDs) which exhibit anti-inflammatory effects that suggest usefulness in diseases such as asthma. How the ability of TZDs to modulate the asthmatic response compares to that of glucocorticoids remains unclear, however, because these two nuclear receptor agonists have never been studied concurrently. Additionally, effects of PPAR-γ agonists have never been examined in a model involving an allergen commonly associated with human asthma.

**Methods:**

We compared the effectiveness of the PPAR-γ agonist pioglitazone (PIO) to the established effectiveness of a glucocorticoid receptor agonist, dexamethasone (DEX), in a murine model of asthma induced by cockroach allergen (CRA). After sensitization to CRA and airway localization by intranasal instillation of the allergen, Balb/c mice were challenged twice at 48-h intervals with intratracheal CRA. Either PIO (25 mg/kg/d), DEX (1 mg/kg/d), or vehicle was administered throughout the period of airway CRA exposure.

**Results:**

PIO and DEX demonstrated similar abilities to reduce airway hyperresponsiveness, pulmonary recruitment of inflammatory cells, serum IgE, and lung levels of IL-4, IL-5, TNF-α, TGF-β, RANTES, eotaxin, MIP3-α, Gob-5, and Muc5-ac. Likewise, intratracheal administration of an adenovirus containing a constitutively active PPAR-γ expression construct blocked CRA induction of Gob-5 and Muc5-ac.

**Conclusion:**

Given the potent effectiveness shown by PIO, we conclude that PPAR-γ agonists deserve investigation as potential therapies for human asthma.

## Background

Asthma incidence and morbidity continues to rise worldwide. Prominent characteristics of allergic asthma include reduced airflow, airway hyperresponsiveness (AHR), accumulation of eosinophils, mast cells, and other inflammatory cells in peribronchiolar regions, and hyperplasia of goblet cells with excessive mucus secretion [1,2]. These effects are accompanied, in part, by the overproduction of a variety of cytokines and chemokines that attract inflammatory cells and stimulate a T_H_2- and IgE-dominated immune response.

Glucocorticoids, inhaled or oral, are currently the most effective treatments for asthma [3]. Side effects remain a significant problem, however, especially since individuals may begin using these medications in childhood and continue them for life. Furthermore, some patients, especially those with severe disease, may respond poorly to steroids or not at all [4]. Consequently, the need remains for medications that are safer and equally or more effective.

Peroxisome proliferator-activated receptors (PPARs) are members of the nuclear hormone receptor superfamily that also includes the glucocorticoid receptor [5]. Members of this family are ligand-activated receptors that classically act by binding to promoter regions of DNA and increasing transcription of specific genes. However they can also interfere with the activity of other transcription factors, such as nuclear factor-κB, and act through pathways unrelated to DNA transcription. The three PPAR isoforms (PPAR-α, PPAR-γ, and PPAR-β/δ) are encoded by separate genes and bind different ligands [6]. Early investigation of PPAR-γ focused on its role in regulating adipocyte differentiation and lipid and glucose metabolism, but more recent studies have demonstrated this receptor's pivotal role in regulation of the immune response [7]. PPAR-γ is now being investigated as a potential target in a variety of lung-related diseases [8].

The synthetic PPAR-γ agonist pioglitazone (PIO), a member of the thiazolidinedione (TZD) drug class, is currently approved for treatment of type 2 diabetes mellitus. PIO and other PPAR-γ ligands have been shown to exert anti-inflammatory effects not only on immune cells [9,10] but also cells specific to the lung such as alveolar macrophages [11], airway epithelial cells [12], and airway smooth muscle cells [13]. Furthermore, PPAR-γ agonists reduce the ability of IL-5 to induce eosinophil survival and chemotaxis [14,15]. These observations suggest that PPAR-γ agonists may prove useful for treatment of inflammatory lung diseases such as asthma [7,16,17]. In contrast to glucocorticoids, PIO demonstrates few side effects.

While previous studies (reviewed in ref. [18]) have demonstrated beneficial effects of PPAR-γ agonists in murine models of asthma, the relevance to human disease of the models employed is unclear. Recent data indicate that exposure to cockroach allergen plays an important role in asthma [19-21]. This finding has led to the development of murine models of human atopic asthma based on sensitization and exposure to cockroach allergen (CRA) [22,23]. CRA challenge results in airway hyperresponsiveness and a robust peribronchial inflammatory response [22]. Since CRA is associated with human asthma, this model appears more clinically applicable as compared with sensitization and challenge with ovalbumin [24]. To our knowledge there have been no studies of PPAR-γ agonists in a murine model of asthma based on exposure to an allergen commonly associated with human airway disease. Furthermore, there does not appear to have been any instance in which effects of PPAR-γ agonists and glucocorticoids were examined concurrently in the same model. The effectiveness of PPAR-γ agonists in asthma consequently remains unclear, especially in comparison to the proven effectiveness of glucocorticoids.

This study directly compares the thiazolidinedione PIO and the glucocorticoid dexamethasone (DEX) in a murine model of asthma induced by CRA. We find that the two compounds have equivalent effects on key pathophysiological responses, cytokine and chemokine levels, and mucus production.

## Methods

### CRA sensitization and challenge

Female Balb/c mice were obtained from Jackson Laboratories (Bar Harbor, ME) and used at 6–8 weeks of age. The mice were sensitized to CRA and challenged as previously described [22]. Briefly, mice were sensitized by intraperitoneal and subcutaneous injection of CRA (Hollister-Stier, Spokane, WA). The response was localized to the airway by intranasal instillation of CRA 14 days later. After another 5 days (day 19 from initial sensitization), mice were anesthetized with an intraperitoneal ketamine and xylazine mixture. Next, the trachea was exposed and mice were challenged by intratracheal (IT) administration of 10 μg of CRA in 50 μl of sterile PBS or were given PBS alone. The skin incision was closed using surgical staples. Mice were then given a second IT CRA or PBS 48 h after the first. All measurements were performed or samples taken 24 h following the second challenge. All experiments were approved by the University of Michigan Committee on Use and Care of Animals.

### PIO and DEX administration

Pioglitazone HCl (kind gift of Hyderabad Biomedical Research Institute, Hyderabad, IN) was dissolved in 0.5% carboxymethylcellulose sodium salt (CMC) (Sigma, St. Louis, MO). Beginning at the time of intranasal instillation of CRA and continuing daily thereafter until the final IT challenge, 25 mg/kg/d of PIO was administered by oral gavage or 1 mg/kg/d of dexamethasone phosphate (Sigma) was administered intraperitoneally. These doses are within the ranges of PIO [25-27] and DEX [28-31] commonly employed in investigations of murine models of various diseases.

### Measurement of airway hyperresponsiveness

AHR was measured as previously described [32] using a plethysmograph (Buxco, Wilmington, NC) that is specifically designed for whole-body measurements on small animals. Briefly, the mouse to be tested was anesthetized with 3.3 mg of sodium pentobarbital (Vortech Pharmaceuticals, Dearborn, MI), and intubated via cannulation of the trachea with an 18-gauge metal tube. The mouse was subsequently ventilated with a Harvard pump ventilator (Harvard Apparatus, Holliston, MA) employing a tidal volume of 0.4 ml, a frequency of 120 breaths/min, and a positive end-respiratory pressure of 2.5–3.0 cm H_2_O. Once anesthesia and ventilation were established, the plethysmograph was sealed and readings monitored by computer. As the box is a closed system, changes in lung volume are reflected in changes of box pressure (P_box_) measured by a differential transducer. After baseline levels had stabilized and initial readings were taken, animals were challenged with nebulized methacholine and the response was monitored. The peak airway resistance was recorded as a measure of airway hyperreactivity.

### Enzyme-linked immunosorbent assays (ELISAs)

The levels of cytokine and chemokine proteins in whole lung homogenate were measured as previously described [22]. Briefly, lung tissue in 1 ml of PBS containing 0.05% Triton X-100 nonionic detergent and antiproteases was homogenized on ice for 30 s with a Tissue Tearor (Biospec Products, Bartlesville, OK). The homogenate was centrifuged at 10,000 × *g *and the resulting supernatant isolated. The murine ELISAs were set up using standardized, specific IL-4, IL-5, TNF-α, RANTES, and eotaxin antibodies (R&D Systems, Minneapolis, MN) that detect protein at concentrations greater than 10 pg/ml and do not crossreact with any other cytokines.

### Serum IgE

Blood was collected from the right ventricle 24 h following the final CRA challenge and centrifuged at 2500 × *g *for 10 min. The serum was then separated and stored at -80°C until analysis. Total serum IgE levels were determined using an IgE ELISA kit (BD BioSciences, San Jose, CA), according to the manufacturer's instructions. Concentrations were calculated using a standard curve generated with the kit's IgE standard.

### Quantitative polymerase chain reaction (PCR) analysis

Total RNA from lung tissues was isolated using TRIzol reagent (Invitrogen, Carlsbad, CA) and chloroform. RNA was quantified by measuring absorption at 260 nm and was stored at -80°C until use. Expression of messenger RNA (mRNA) was determined by real-time reverse transcriptase polymerase chain reaction (RT-PCR) using the ABI Prism 7700 Detection System (TaqMan; Applied Biosystems, Foster City, CA). Primers and probes were designed using Primer Express software (Applied Biosystems) and are shown in Table 1.

**Table 1:** Primers and probes used for real-time PCR of mouse lung RNA

Gene	Primer/Probe Sequences
*Gob-5*	
forward	5' GAGTGGGCTCACTTCCGATG 3'
reverse	5' GCTGAACACCTCACTGCTTGG 3'
Probe	5' CAACAACGACGAGAAGTTCTACTTATCCAAAGG 3'
*Muc5-ac*	
forward	5' CCAGCACCATCTCTACAACCC 3'
reverse	5' GCAAAGCTCCTGTTTGCACTC 3'
Probe	5' CCCAAACTATCTCAACCTCAGGGTCCACC 3'
*MIP3-α*	
forward	5' CCTTGCTTTGGCATGGGTACT 3'
reverse	5' TCGTAGTTGTTGCTGTTCTG 3'
Probe	5' CTGGCTCACCTCTGCAGCCAG 3'
*TGF-β*	
forward	5' GACCCTGCCCCTATATTTGGA 3'
reverse	5' CGCCCGGGTTGTGTTG 3'
Probe	5' CACAGTACAGCAAGGTCCTTGCCCTCTACA 3'

### Microscopic examination of lung tissue

Twenty-four h following the final CRA challenge, lungs were perfused and fixed for 10 h with 10% (v/v) neutral buffered formalin, then transferred to 75% ethanol. After fixation, lung tissues were embedded in paraffin, and 5 μm sections were routinely processed and stained with H&E for light microscopic analysis. To estimate the number of eosinophils in the peribronchial region, these cells were counted in 100 high-powered fields per lung as described previously [22]. Other sections were stained with periodic acid Schiff's (PAS) to examine the extent of mucin production.

### Construction of adenoviral vector containing constitutively active PPAR-γ expression plasmid

VP16-PPAR-γ2, in which the potent viral transcriptional activator VP16 is fused to PPAR-γ2, constitutively activates PPAR-γ-responsive genes in the absence of ligands [33]. The adenoviral vector AdCMV-VP16-PPAR-γ2 was constructed by isolation of VP16-PPAR-γ2 through digestion of pCMX-VP16-PPAR-γ2 (kind gift of Dr. Mitchell Lazar, University of Pennsylvania, Philadelphia, PA) with the restriction enzymes SpeI and NheI. The VP16-PPAR-γ2 fragment was then inserted into the SpeI/XbaI site of the Ad5 shuttle vector pACCMV2. A full-length E1, E3 deleted recombinant adenovirus was generated using *in situ *loxP recombination between the shuttle vector (linearized with PmeI) and the cAd5-deltaE3.LoxP cosmid containing the Ad5 backbone (linearized with ClaI) in the presence of purified Cre recombinase [34]. The resulting recombinant adenoviral DNA was then transfected into HER 911 cells by standard calcium phosphate precipitation methods. Recombinant clones were identified as plaques in soft agar culture and the presence of functional VP16-PPAR-γ2 was verified by infecting A549 cells expressing a PPAR-dependent luciferase reporter construct (pFATP-luc) [35]. Large scale, high titer adenoviral purification, particle determination (particles/ml) and titer determination (plaque forming units/ml) were performed by the University of Michigan Vector Core. Aliquots of AdCMV-VP16-PPAR-γ2 were maintained at -80°C until immediately prior to use.

### Intratracheal administration of AdCMV-VP16-PPAR-γ2

Constitutively active PPAR-γ expression construct was delivered to mice by IT administration of 1 × 10^9 ^pfu of AdCMV-VP16-PPAR-γ2 at the time of intranasal administration of CRA and again 5 d later, at the time of IT CRA challenge. Control mice received empty adenoviral vector at the same times.

### Statistical analysis

Values were expressed as means ± SEMs. Data were analyzed with one-way ANOVAs with Bonferroni paired-group planned comparisons and Kruskal-Wallis tests with Dunn paired-group planned comparisons using Prism 5 software (GraphPad Software, San Diego, CA). Statistical significance was defined as *P *< 0.05.

## Results

### PIO inhibits key pathophysiological responses to CRA challenge as effectively as DEX

**Figure 1:**
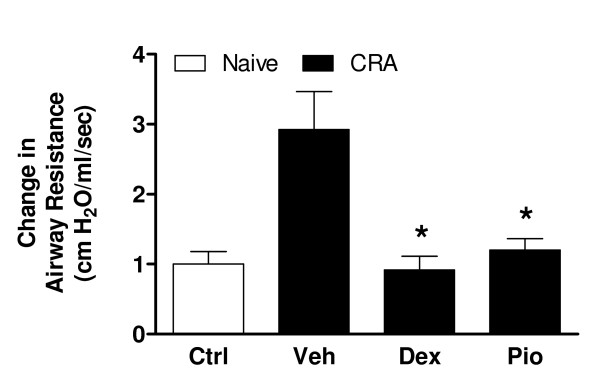
**PIO decreases airway resistance in CRA challenged mice as effectively as DEX**. PIO, DEX, or vehicle (Veh) was administered daily (days 14–21). Airway response to challenge with nebulized methacholine was measured in anesthetized mice (8 mice/group) using a plethysmograph specifically designed for whole-body measurements on small animals. Measurements were performed 24 h following final CRA challenge. *P < 0.05 compared to vehicle treatment.

**Figure 2:**
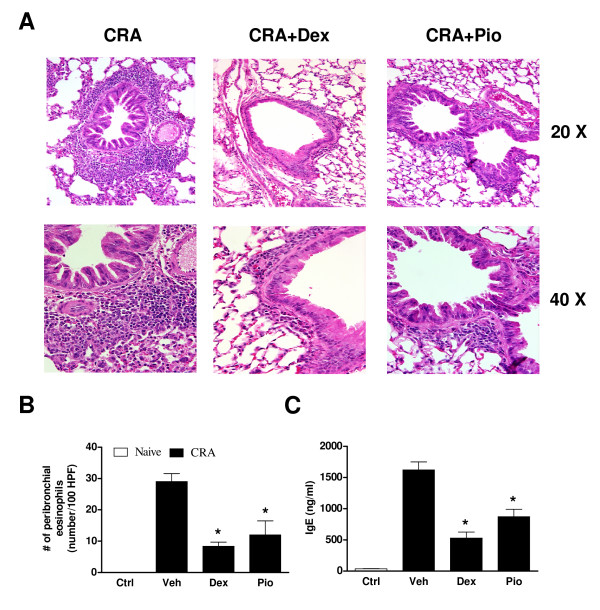
**PIO inhibits inflammatory responses to CRA challenge as effectively as DEX**. PIO, DEX, or vehicle (Veh) was administered daily (days 14–21). All measurements (8 mice/group) were performed 24 h following final CRA challenge. **A**. Peribronchial inflammatory cell infiltration was visualized by H&E staining. **B**. Eosinophil infiltration was quantified by counting 100 high-powered fields per lung. **C**. Total serum IgE levels were measured by ELISA. *P < 0.05 compared to vehicle treatment.

Clinically, airway hyperresponsiveness is a prominent feature of asthma. We found that the increased airway resistance in response to methacholine challenge was significantly greater in CRA-challenged mice than in control animals (*P *< 0.05) but was abolished with either PIO (*P *< 0.05) or DEX (Fig. 1; *P *< 0.05). Histologically, asthma is characterized by infiltration of peribronchial tissues with inflammatory cells, in particular eosinophils. Eosinophils are both major sources of proinflammatory mediators and effector cells in airway remodeling. Abundant peribronchial infiltration of leukocytes was seen in H&E-stained sections from lungs of vehicle-treated mice with infiltration of these cells greatly reduced in mice treated with PIO or DEX (Fig. 2A). Likewise, morphometric analysis demonstrated a significant decrease in the number of eosinophils present following PIO (*P *< 0.05) or DEX (*P *< 0.05) treatment (Fig. 2B). Excessive levels of serum IgE directed against the sensitizing antigen, with consequently an inappropriately exuberant cell-mediated response to subsequent antigen exposure, are also typical of allergic asthma. Serum IgE was found to be low (~40 ng/ml) in control mice but was present at levels exceeding 1500 ng/ml in those challenged with CRA (Fig. 2C; *P *< 0.05). Levels were significantly lower in mice treated with either nuclear hormone receptor agonist (*P *< 0.05).

### PIO reduces pulmonary cytokine and chemokine levels as effectively as DEX

**Figure 3:**
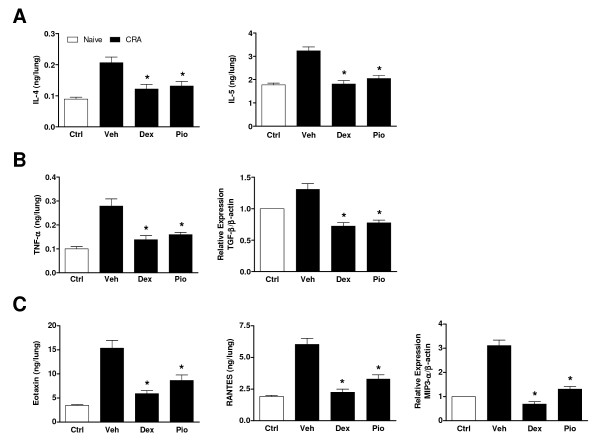
**PIO reduces pulmonary cytokine and chemokine levels as effectively as DEX**. PIO, DEX, or vehicle (Veh) was administered daily (days 14–21). Twenty-four h following the final CRA challenge, cytokine levels were measured (by ELISA unless otherwise noted) in homogenized lung tissue from 8 mice/group. **A**. T_H_2 cytokines IL-4 and IL-5. **B **Cytokines TNF-α and TGF-β. TGF-β expression was measured using quantitative PCR analysis of total RNA from lung tissue. The ratio of TGF-β expression to β-actin expression in control animals was assigned a value of 1. **C**. Chemokines eotaxin, RANTES, and MIP3-α. MIP3-α expression was measured using quantitative PCR analysis of total RNA from lung tissue. The ratio of MIP3-α expression to β-actin expression in control animals was assigned a value of 1. *P < 0.05 compared to vehicle treatment.

Overproduction of a variety of cytokines and chemokines is characteristic of asthma pathophysiology. CRA challenge similarly resulted in induction of lung cytokines and chemokines. These included the T_H_2 cytokines IL-4 (*P *< 0.05) and IL-5 (*P *< 0.05), whose actions include, respectively, class switching to IgE production and eosinophil activation (Fig. 3A). Levels of the proinflammatory cytokine tumor necrosis factor-α (TNF-α; *P *< 0.05), which promotes inflammation, mucus secretion, and airway hyperresponsiveness, were likewise increased (Fig. 3B). The chemokines eotaxin (*P *< 0.05) and RANTES (*P *< 0.05), which are primarily responsible for recruitment of eosinophils, and MIP3-α (*P *< 0.05), a chemokine that attracts memory T-cells and immature dendritic cells, were also significantly elevated compared to control animals (Fig. 3C). In each case, increases in cytokines and chemokines were blunted to a similar degree with PIO (*P *< 0.05) or DEX (*P *< 0.05) treatment. While CRA induced only a modest increase in expression of mRNA for the profibrotic cytokine transforming growth factor-β (TGF-β), expression in animals treated with PIO (*P *< 0.05) or DEX (*P *< 0.05) was still significantly reduced (Fig. 3B).

### PIO, DEX, or AdCMV-VP16-PPARγ2 effectively reduces mucin production and Gob-5 and Muc5-ac mRNA expression

**Figure 4:**
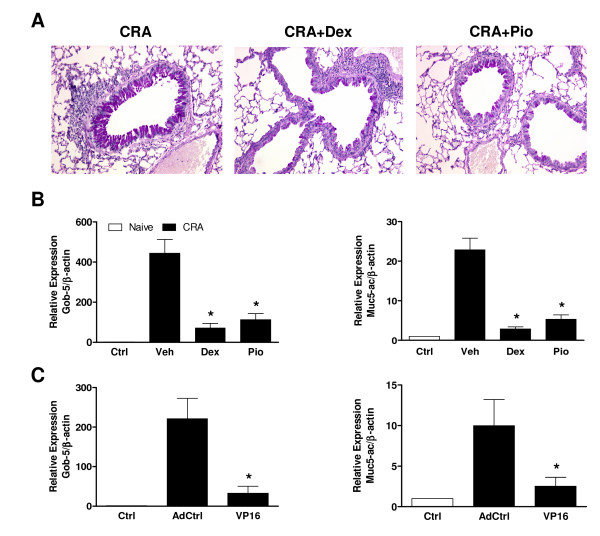
**PIO, DEX, or constitutively active PPAR-γ effectively reduces mucin production and *Gob-5 *and *Muc5-ac *expression**. PIO, DEX, or vehicle (Veh) was administered daily (days 14–21). All measurements were carried out 24 h following the final CRA challenge. **A**. Lung tissue (8 mice/group) was fixed and stained with periodic acid Schiff's (PAS) to examine the extent of mucin production. **B**. Quantitative PCR was used to measure expression of Gob-5 and Muc5-ac mRNA (8 mice/group) from lung tissue. For each gene, the control-group ratio of its expression to that of β-actin was assigned a value of 1. *P < 0.05 compared to vehicle treatment. **C**. AdCMV-VP16-PPAR-γ2 (VP16) or an empty control vector (AdCtrl) was administered as described in Materials and Methods. Quantitative PCR was used to measure expression of Gob-5 and Muc5-ac mRNA (5 mice/group) from lung tissue as mentioned. Effect on mucin production was also observed (data not shown). *P < 0.05 compared to AdCtrl administration.

Asthma is associated with mucus overproduction, resulting in airway narrowing and obstructive lung disease. While goblet cell hyperplasia and mucin production were readily seen in PAS-stained lung sections from CRA-challenged animals treated with vehicle, they were much less apparent in those from PIO- or DEX-treated animals (Fig. 4A). No positive staining for PAS was observed in control lung sections (data not shown). Goblet cell hyperplasia and mucin production are associated with increased transcription of the *Gob-5 *and *Muc5-ac *genes [24,36]. Little expression of either Gob-5 or Muc5-ac mRNA was observed in control mice, but levels more than 500 (Gob-5; *P *< 0.05) or 20 (Muc5-ac; *P *< 0.05) times greater were seen in CRA-challenged mice (Fig. 4B). These elevations in mRNA levels were dramatically reduced by treatment with either PIO (*P *< 0.05) or DEX (*P *< 0.05). Similarly, when mice were intratracheally administered an adenoviral vector containing an expression plasmid generating a constitutively active form of PPAR-γ, AdCMV-VP16-PPAR-γ2, the ability of CRA to induce expression of Gob-5 and Muc5-ac was significantly suppressed (*P *< 0.05) compared to mice given control adenovirus (Fig. 4C). AdCMV-VP16-PPAR-γ2 treated mice also demonstrated inhibition of goblet cell hyperplasia and mucin production (data not shown).

## Discussion

In this study we found that both PIO and DEX significantly reduce or eliminate many of the allergen-induced responses in a murine model of acute asthma induced by CRA sensitization and challenge. Specifically, we observed major decreases in airway hyperresponsiveness, peribronchial infiltration of inflammatory cells, and lung cytokine and chemokine levels with PIO or DEX treatment. Similar reductions were seen in goblet cell hyperplasia and mucin production along with expression of the *Gob-5 *and *Muc5-ac *genes. The consistent similarity between effects of PIO and those of DEX is striking. In every aspect examined, these two nuclear hormone receptor agonists produced similar, if not identical, reductions in the response to CRA challenge. In many cases, values were reduced to those seen in control animals.

We also show, for the fist time, that induction of Gob-5 and Muc5-ac expression was blocked in the presence of a constitutively active PPAR-γ (VP16-PPAR-γ2) delivered intratracheally to mice via an adenoviral vector. This observation suggests that the modulation of mucus-associated genes occurs in a PPAR-γ-specific manner. Previous studies in other model systems have employed adenoviral delivery of a PPAR-γ cDNA expression plasmid [37-39]. These studies did not examine effects on Gob-5 and Muc5-ac expression but found that overexpression of PPAR-γ affected other markers of inflammation and remodeling similarly to PPAR-γ agonists. Since these effects were seen in the absence of exogenous agonists, they presumably reflect activation of PPAR-γ by endogenous ligands that would be up-regulated during the asthmatic response.

Previous studies of PPAR-γ agonists in murine models of asthma have been based either on sensitization and challenge with ovalbumin [36-38,40-43] or on treatment with toluene diisocyanate [39]. Our results, however, were obtained in a model utilizing an allergen relevant to human asthma [19-21]. Furthermore, none of the previous studies have concurrently examined the effects of glucocorticoids. Thus, this present study not only extends knowledge of PPAR-γ agonists' beneficial effects to an asthma model employing an allergen known to be associated with human disease, but for the first time allows a direct comparison between the effectiveness of PPAR-γ agonists and that of glucocorticoids.

Our results are consistent with those obtained in other asthma models. Studies using PPAR-γ agonists have found reductions in inflammation [15,37-39,41-43] and in airway hyperresponsiveness [15,37-41]. Choices of other aspects of asthma for study have varied widely. Several studies reported a reduction in mucus production [39,40,42] with TZD treatment. Decreased levels of asthma-associated cytokines, and especially of the T_H_2 cytokine IL-4, have also been frequently reported [15,38,39,41,42]. Decreased expression of chemokines has likewise been seen in other models [38,39], although one study reported that ciglitazone had no effect on eotaxin levels [15]. Our study is the first to demonstrate PPAR-γ-induced downregulation of the *Gob-5 *and *Muc5-ac *genes.

This also is the first study to demonstrate the beneficial effects of dexamethasone in the CRA-induced murine model of asthma. Previous studies had examined the effects of dexamethasone in the ovalbumin model [28,44-55] as well as in an asthma model induced by house dust [30] and in mice with constitutive bronchial hyperresponsiveness [56]. Most of these, however, either focused on fibrosis and airway remodeling or examined mechanistic aspects of the response.

PPAR-γ ligands have been shown to exert effects on a variety of cells of the immune system including macrophages [9,57], neutrophils [58], dendritic cells [59], B [60] and T [61-63] lymphocytes, eosinophils [15], natural killer cells [64], and mast cells [10]. A number of mechanisms have been proposed by which PPAR-γ agonists inhibit detrimental asthmatic responses, including interference with actions of nuclear factor-κB [37], upregulation of phosphatase and tensin homolog deleted on chromosome 10 (PTEN) [38], and upregulation of IL-10 [41]. However, aside from the demonstration that antibody to the IL-10 receptor partially blocked effects of rosiglitazone, these suggestions are based solely on observed correlation.

As shown by our failure to see a statistically significant increase in the profibrotic cytokine TGF-β, the model of acute asthma employed in our study does not address such long-term effects of asthma as fibrosis and airway remodeling. However, a CRA-induced model of chronic asthma, based on multiple allergen challenges, has been developed [65]. PPAR-γ agonists have not to date been investigated in this model. Nevertheless, such agents have been shown to inhibit airway smooth muscle cell proliferation and induce apoptosis of these cells *in vitro *[13] and to inhibit various aspects of airway remodeling in the toluene diisocyanate [39] and ovalbumin models [40]. Notably, the PPAR-γ agonist in this latter study was administered by inhalation, demonstrating that this is an effective route of administration that allows these compounds to be used in the same manner as inhaled glucocorticoids.

Another possibility deserving of further investigation is that activation of PPAR-γ and the glucocorticoid receptor may result in synergistic effects. The possibility of synergism is strengthened by the observation that dexamethasone upregulates PPAR-γ expression in eosinophils [66]. Furthermore, it has been reported that PPAR-γ agonists and glucocorticoids additively inhibit TNF-α-induced production of eotaxin and monocyte chemotactic protein-1 by airway smooth muscle cells [67]. Should synergy be demonstrated, then optimal effects could be obtained by combining low and therefore relatively safer doses of a glucocorticoid with an appropriate PPAR-γ agonist.

## Conclusion

In conclusion, we find that on a wide variety of measures PIO, like DEX, greatly reduces or eliminates response to CRA challenge in a murine model of asthma. Any differences in effectiveness between the two compounds were minor. To our knowledge this is not only the first study to investigate PIO in a model using an allergen associated with human disease but also the first time any model has been used to examine a PPAR-γ agonist and a glucocorticoid concurrently. Since the clinical safety of PIO has been demonstrated, and since our results indicate that its effectiveness in a murine model of asthma is similar to that of glucocorticoids, we suggest that it should be investigated as a potential therapy for human asthma.

## Competing interests

The author(s) declare that they have no competing interests.

## Authors' contributions

VRN performed the majority of the studies, participated in study design and data interpretation, and drafted the manuscript. RR constructed the AdCMV-VP16-PPAR-γ2. MRS and AAB carried out the cytokine and chemokine ELISAs. NWL and TJS participated in the design of the study. RCR provided input and oversight regarding all aspects of study design and interpretation of results. He was also responsible for revising and finalizing the manuscript. All authors read and approved the final manuscript.

## References

[B1] Wills-Karp M (1999). Immunologic basis of antigen-induced airway hyperresponsiveness. Annu Rev Immunol.

[B2] Ying S, Zhang G, Gu S, Zhao J (2006). How much do we know about atopic asthma: where are we now?. Cell Mol Immunol.

[B3] Barnes PJ (1995). Inhaled glucocorticoids for asthma. N Engl J Med.

[B4] Mjaanes CM, Whelan GJ, Szefler SJ (2006). Corticosteroid therapy in asthma: predictors of responsiveness. Clin Chest Med.

[B5] Glass CK, Ogawa S (2006). Combinatorial roles of nuclear receptors in inflammation and immunity. Nat Rev Immunol.

[B6] Chinetti G, Fruchart JC, Staels B (2000). Peroxisome proliferator-activated receptors (PPARs): nuclear receptors at the crossroads between lipid metabolism and inflammation. Inflamm Res.

[B7] Becker J, Delayre-Orthez C, Frossard N, Pons F (2006). Regulation of inflammation by PPARs: a future approach to treat lung inflammatory diseases?. Fundam Clin Pharmacol.

[B8] Huang TH-W, Razmovski-Naumovski V, Kota BP, Lin DS-H, Roufogalis BD (2005). The pathophysiological function of peroxisome proliferator-activated receptor-γ in lung-related diseases. Respir Res.

[B9] Jiang C, Ting AT, Seed B (1998). PPAR-γ agonists inhibit production of monocyte inflammatory cytokines. Nature.

[B10] Sugiyama H, Nonaka T, Kishimoto T, Komoriya K, Tsuji K, Nakahata T (2000). Peroxisome proliferator-activated receptors are expressed in human cultured mast cells: a possible role of these receptors in negative regulation of mast cell activation. Eur J Immunol.

[B11] Reddy RC, Keshamouni VG, Jaigirdar SH, Zeng X, Leff T, Thannickal VJ, Standiford TJ (2004). Deactivation of murine alveolar macrophages by peroxisome proliferator-activated receptor-γ ligands. Am J Physiol Lung Cell Mol Physiol.

[B12] Wang ACC, Dai X, Luu B, Conrad DJ (2001). Peroxisome proliferator-activated receptor-γ regulates airway epithelial cell activation. Am J Respir Cell Mol Biol.

[B13] Patel HJ, Belvisi MG, Bishop-Bailey D, Yacoub MH, Mitchell JA (2003). Activation of peroxisome proliferator-activated receptors in human airway smooth muscle cells has a superior anti-inflammatory profile to corticosteroids: relevance for chronic obstructive pulmonary disease therapy. J Immunol.

[B14] Ueki S, Usami A, Oyamada H, Saito N, Chiba T, Mahemuti G, Ito W, Kato H, Kayaba H, Chihara J (2006). Procaterol upregulates peroxisome proliferator-activated receptor-γ expression in human eosinophils. Int Arch Allergy Immunol.

[B15] Woerly G, Honda K, Loyens M, Papin JP, Auwerx J, Staels B, Capron M, Dombrowicz D (2003). Peroxisome proliferator-activated receptors α and γ down-regulate allergic inflammation and eosinophil activation. J Exp Med.

[B16] Denning GM, Stoll LL (2006). Peroxisome proliferator-activated receptors: potential therapeutic targets in lung disease?. Pediatr Pulmonol.

[B17] Spears M, McSharry C, Thomson NC (2006). Peroxisome proliferator-activated receptor-γ agonists as potential anti-inflammatory agents in asthma and chronic obstructive pulmonary disease. Clin Exp Allergy.

[B18] Belvisi MG, Hele DJ, Birrell MA (2006). Peroxisome proliferator-activated receptor gamma agonists as therapy for chronic airway inflammation. Eur J Pharmacol.

[B19] Arruda LK, Vailes LD, Platts-Mills TAE, Hayden ML, Chapman MD (1997). Induction of IgE antibody responses by glutathione *S*-transferase from the German cockroach (*Blattella germanica*). J Biol Chem.

[B20] Kang B, Vellody D, Homburger H, Yunginger JW (1979). Cockroach cause of allergic asthma. Its specificity and immunologic profile. J Allergy Clin Immunol.

[B21] Rosenstreich DL, Eggleston P, Kattan M, Baker D, Slavin RG, Gergen P, Mitchell H, McNiff-Mortimer K, Lynn H, Ownby D (1997). The role of cockroach allergy and exposure to cockroach allergen in causing morbidity among inner-city children with asthma. N Engl J Med.

[B22] Campbell EM, Kunkel SL, Strieter RM, Lukacs NW (1998). Temporal role of chemokines in a murine model of cockroach allergen-induced airway hyperreactivity and eosinophilia. J Immunol.

[B23] Sarpong SB, Zhang LY, Kleeberger SR (2003). A novel mouse model of experimental asthma. Int Arch Allergy Immunol.

[B24] Kung TT, Jones H, Adams GK, Umland SP, Kreutner W, Egan RW, Chapman RW, Watnick AS (1994). Characterization of a murine model of allergic pulmonary inflammation. Int Arch Allergy Immunol.

[B25] Akahori T, Sho M, Hamada K, Suzaki Y, Kuzumoto Y, Nomi T, Nakamura S, Enomoto K, Kanehiro H, Nakajima Y (2007). Importance of peroxisome proliferator-activated receptor-gamma in hepatic ischemia/reperfusion injury in mice. J Hepatol.

[B26] Kubota N, Terauchi Y, Kubota T, Kumagai H, Itoh S, Satoh H, Yano W, Ogata H, Tokuyama K, Takamoto I (2006). Pioglitazone ameliorates insulin resistance and diabetes by both adiponectin-dependent and – independent pathways. J Biol Chem.

[B27] Srinivasan K, Patole PS, Kaul CL, Ramarao P (2004). Reversal of glucose intolerance by by pioglitazone in high fat diet-fed rats. Methods Find Exp Clin Pharmacol.

[B28] Blyth DI, Wharton TF, Pedrick MS, Savage TJ, Sanjar S (2000). Airway subepithelial fibrosis in a murine model of atopic asthma: suppression by dexamethasone or anti-interleukin-5 antibody. Am J Respir Cell Mol Biol.

[B29] Christie PE, Jonas M, Tsai C-H, Chi E-Y, Henderson WR (2004). Increase in laminin expression in allergic airway remodelling and decrease by dexamethasone. Eur Respir J.

[B30] Kim J, McKinley L, Siddiqui J, Bolgos GL, Remick DG (2004). Prevention and reversal of pulmonary inflammation and airway hyperresponsiveness by dexamethasone treatment in a murine model of asthma induced by house dust. Am J Physiol Lung Cell Mol Physiol.

[B31] Roh GS, Shin Y, Seo S-W, Yoon BR, Yeo S, Park SJ, Cho JW, Kwack K (2004). Proteome analysis of differential protein expression in allergen-induced asthmatic mice lung after dexamethasone treatment. Proteomics.

[B32] Lukacs NW, Strieter RM, Warmington K, Lincoln P, Chensue SW, Kunkel SL (1997). Differential recruitment of leukocyte populations and alteration of airway hyperreactivity by C-C family chemokines in allergic airway inflammation. J Immunol.

[B33] Li Y, Lazar MA (2002). Differential gene regulation by PPARγ agonist and constitutively active PPARγ2. Mol Endocrinol.

[B34] Aoki K, Barker C, Danthinne X, Imperiale MJ, Nabel GJ (1999). Efficient generation of recombinant adenoviral vectors by Cre-lox recombination in vitro. Mol Med.

[B35] Keshamouni VG, Reddy RC, Arenberg DA, Joel B, Thannickal VJ, Kalemkerian GP, Standiford TJ (2004). Peroxisome proliferator-activated receptor-γ activation inhibits tumor progression in non-small-cell lung cancer. Oncogene.

[B36] Hammad H, de Heer HJ, Soullié T, Angeli V, Trottein F, Hoogsteden HC, Lambrecht BN (2004). Activation of peroxisome proliferator-activated receptor-γ in dendritic cells inhibits the development of eosinophilic airway inflammation in a mouse model of asthma. Am J Pathol.

[B37] Lee KS, Kim SR, Park SJ, Park HS, Min KH, Jin SM, Lee MK, Kim UH, Lee YC (2006). Peroxisome proliferator activated receptor-γ modulates reactive oxygen species generation and activation of nuclear factor-κB and hypoxia-inducible factor 1α in allergic airway disease of mice. J Allergy Clin Immunol.

[B38] Lee KS, Park SJ, Hwang PH, Yi HK, Song CH, Chai OH, Kim JS, Lee MK, Lee YC (2005). PPAR-gamma modulates allergic inflammation through up-regulation of PTEN. FASEB J.

[B39] Lee KS, Park SJ, Kim SR, Min KH, Jin SM, Lee HK, Lee YC (2006). Modulation of airway remodeling and airway inflammation by peroxisome proliferator-activated receptor γ in a murine model of toluene diisocyanate-induced asthma. J Immunol.

[B40] Honda K, Marquillies P, Capron M, Dombrowicz D (2004). Peroxisome proliferator-activated receptor γ is expressed in airways and inhibits features of airway remodeling in a mouse asthma model. J Allergy Clin Immunol.

[B41] Kim SR, Lee KS, Park HS, Park SJ, Min KH, Jin SM, Lee YC (2005). Involvement of IL-10 in peroxisome proliferator-activated receptor γ-mediated anti-inflammatory response in asthma. Mol Pharmacol.

[B42] Mueller C, Weaver V, Vanden Heuvel JP, August A, Cantorna MT (2003). Peroxisome proliferator-activated receptor γ ligands attenuate immunological symptoms of experimental allergic asthma. Arch Biochem Biophys.

[B43] Trifilieff A, Bench A, Hanley M, Bayley D, Campbell E, Whittaker P (2003). PPAR-α and -γ but not -δ agonists inhibit airway inflammation in a murine model of asthma:*in vitro *evidence for an NF-κB-independent effect. Br J Pharmacol.

[B44] Birrell MA, Battram CH, Woodman P, McCluskie K, Belvisi MG (2003). Dissociation by steroids of eosinophilic inflammation from airway hyperresponsiveness in murine airways. Respir Res.

[B45] Christie PE, Jonas M, Tsai C-H, Chi EY, Henderson WR (2004). Increase in laminin expression in allergic airway remodelling and decrease by dexamethasone. Eur Respir J.

[B46] De Bie JJ, Hessel EM, Van Ark I, Van Esch B, Hofman G, Nijkamp FP, Van Oosterhout AJ (1996). Effect of dexamethasone and endogenous corticosterone on airway hyperresponsiveness and eosinophilia in the mouse. Br J Pharmacol.

[B47] Eum S-Y, Maghni K, Hamid Q, Eidelman DH, Campbell H, Isogai S, Martin JG (2003). Inhibition of allergic airways inflammation and airway hyperresponsiveness in mice by dexamethasone: role of eosinophils, IL-5, eotaxin, and IL-13. J Allergy Clin Immunol.

[B48] Jungsuwadee P, Dekan G, Stingl G, Epstein MM (2004). Inhaled dexamethasone differentially attenuates disease relapse and established allergic asthma in mice. Clin Immunol.

[B49] Kumar RK, Herbert C, Thomas PS, Wollin L, Beume R, Yang M, Webb DC, Foster PS (2003). Inhibition of inflammation and remodeling by roflumilast and dexamethasone in murine chronic asthma. J Pharmacol Exp Ther.

[B50] Kurokawa M, Kokubu F, Matsukura S, Kawaguchi M, Ieki K, Suzuki S, Odaka M, Watanabe S, Takeuchi H, Akabane T (2005). Effects of corticosteroid on the expression of thymus and activation-regulated chemokine in a murine model of allergic asthma. Int Arch Allergy Immunol.

[B51] Miller M, Cho JY, McElwain K, McElwain S, Shim JY, Manni M, Baek JS, Broide DH (2006). Corticosteroids prevent myofibroblast accumulation and airway remodeling in mice. Am J Physiol Lung Cell Mol Physiol.

[B52] Sun J-g, Deng Y-m, Wu X, Tang H-f, Deng J-f, Chen J-q, Yang S-y, Xie Q-m (2006). Inhibition of phosphodiesterase activity, airway inflammation and hyperresponsiveness by PDE4 inhibitor and glucocorticoid in a murine model of allergic asthma. Life Sci.

[B53] Trifilieff A, El-Hashim A, Bertrand C (2000). Time course of inflammatory and remodeling events in a murine model of asthma: effect of steroid treatment. Am J Physiol Lung Cell Mol Physiol.

[B54] Zhang Y, Adner M, Cardell L-O (2005). Glucocorticoids suppress transcriptional up-regulation of bradykinin receptors in a murine in vitro model of chronic airway inflammation. Clin Exp Allergy.

[B55] Zhao J, Yeong LH, Wong WS (2007). Dexamethasone alters bronchoalveolar lavage fluid proteome in a mouse asthma model. Int Arch Allergy Immunol.

[B56] Eum S-Y, Creminon C, Haile S, Lefort J, Vargaftig BB (1996). Inhibition of airways inflammation by dexamethasone is followed by reduced bronchial hyperreactivity in BP2 mice. Clin Exp Allergy.

[B57] Ricote M, Li AC, Willson TM, Kelly CJ, Glass CK (1998). The peroxisome proliferator-activated receptor-γ is a negative regulator of macrophage activation. Nature.

[B58] Standiford TJ, Keshamouni VG, Reddy RC (2005). Peroxisome proliferator-activated receptor-γ as a regulator of lung inflammation and repair. Proc Am Thorac Soc.

[B59] Nencioni A, Grunebach F, Zobywlaski A, Denzlinger C, Brugger W, Brossart P (2002). Dendritic cell immunogenicity is regulated by peroxisome proliferator-activated receptor γ. J Immunol.

[B60] Padilla J, Kaur K, Cao HJ, Smith TJ, Phipps RP (2000). Peroxisome proliferator activator receptor-γ agonists and 15-Deoxy-Δ^12,14^-PGJ_2 _induce apoptosis in normal and malignant B-lineage cells. J Immunol.

[B61] Clark RB, Bishop-Bailey D, Estrada-Hernandez T, Hla T, Puddington L, Padula SJ (2000). The nuclear receptor PPARγ and immunoregulation: PPARγ mediates inhibition of helper T cell responses. J Immunol.

[B62] Cunard R, Eto Y, Muljadi JT, Glass CK, Kelly CJ, Ricote M (2004). Repression of IFN-γ expression by peroxisome proliferator-activated receptor γ. J Immunol.

[B63] Yang XY, Wang LH, Chen T, Hodge DR, Resau JH, DaSilva L, Farrar WL (2000). Activation of human T lymphocytes is inhibited by peroxisome proliferator-activated receptor γ (PPARγ) agonists. PPARγ co-association with transcription factor NFAT. J Biol Chem.

[B64] Zhang X, Rodriguez-Galan MC, Subleski JJ, Ortaldo JR, Hodge DL, Wang JM, Shimozato O, Reynolds DA, Young HA (2004). Peroxisome proliferator-activated receptor-γ and its ligands attenuate biologic functions of human natural killer cells. Blood.

[B65] Berlin AA, Hogaboam CM, Lukacs NW (2006). Inhibition of SCF attenuates peribronchial remodeling in chronic cockroach allergen-induced asthma. Lab Invest.

[B66] Usami A, Ueki S, Ito W, Kobayashi Y, Chiba T, Mahemuti G, Oyamada H, Kamada Y, Fujita M, Kato H (2006). Theophylline and dexamethasone induce peroxisome proliferator-activated receptor-γ expression in human eosinophils. Pharmacology.

[B67] Nie M, Corbett L, Knox AJ, Pang L (2005). Differential regulation of chemokine expression by peroxisome proliferator-activated receptor γ agonists: interactions with glucocorticoids and β_2_-agonists. J Biol Chem.

